# Preparation of Waste-LDPE/SBS Composite High-Viscosity Modifier and Its Effect on the Rheological Properties and Microstructure of Asphalt

**DOI:** 10.3390/polym14183848

**Published:** 2022-09-14

**Authors:** Haosheng Yu, Yong Jin, Xingmin Liang, Fuqiang Dong

**Affiliations:** 1 Nanjing Foreign Language School Xianlin Campus, Nanjing 210023, China; 2 College of Civil and Transportation Engineering, Hohai University, Nanjing 210098, China

**Keywords:** white pollution, environment-friendly high-viscosity modifier, physical characteristics, rheological performance, microstructure

## Abstract

To reduce the cost of high-viscosity modifier (HVM) and alleviate white pollution problems, we prepared the environment-friendly HVM (E-HVM) by using waste-low density polyethylene/styrene-butadiene-styrene (waste-LDPE/SBS) composite. The physical characteristics of the E-HVM modifier were first investigated. Additionally, the effects of E-HVM modifier dosage (8 wt% to 20 wt%) on the rheological properties and microstructure of asphalt were, respectively, researched by dynamic shear rheometer (DSR), bending beam rheometer (BBR), and fluorescence microscopy (FM). The results show that the E-HVM modifier has lower molecular weight, and its distribution is wider than that of the Tafpack-Super (TPS) modifier; thus, the E-HVM modifier had better compatibility with asphalt, which has also been proven by FM images. Due to these reasons, the E-HVM modifier improves the high-temperature performances of asphalt more effectively than the TPS modifier, which is shown by the higher dynamic viscosity (60 °C) and G^*^ and the lower δ and $J_{\mathrm{nr}}\left(\tau \right)$ Furthermore, compared to TPS modified asphalt, E-HVM modified asphalt also has a higher fatigue life at different strain levels (2.5% and 5.0%), but worse low-temperature performance. Following a comprehensive consideration of performances, the reasonable dosage range of E-HVM modifier is 12 wt% to 16 wt%.

## 1. Introduction

The excellent drainage performance and noise-reduction effect of porous asphalt pavement have caught the attention of road researchers [1,2]. Compared with traditional dense asphalt pavement, porous asphalt pavement has better anti-slide performance and reduction of noise. However, asphalt mixtures with high porosities are also less durable and shear resistant [3,4,5]. This means that porous asphalt pavement requires asphalt with excellent viscosity and toughness and aging resistance, as well as good adhesion properties to the aggregates [6,7]. Therefore, the ordinary modified asphalt binder is not able to meet the performance requirements of porous asphalt pavements, while the high-viscosity asphalt (HVA) is the preferred asphalt [8]. The dynamic viscosity of HVA can reach tens of times that of ordinary modified asphalt, which makes it more effective for enhancing aggregate bonding. The modified asphalt with dynamic viscosity (60 °C) greater than 20,000 Pa·s is defined as HVA in Japan, and it has been extensively used [9]. Zhang et al. [10] found that asphalt had better high-temperature performances and stronger holding power to aggregate when its dynamic viscosity (60 °C) was higher. Therefore, HVA plays an important role in determining the performance of porous asphalt pavement [11]. Modifiers and asphalt are cross-linked chemically or physically to achieve HVA. The performance of HVA is affected by different modifiers, so the quality of the modifier is crucial.

Currently, there are three main categories of modifiers applied to drainage pavement: thermoplastic elastomers, rubber, and high-viscosity modifiers (HVM). Luo et al. [12] found the HVA modified with a 6 wt% styrene–butadiene–styrene (SBS) composite modifier (SBS1301:SBS4303 = 2:1) shows similar performance to SinoTPS modified asphalt and Tafpack-Super (TPS) modified asphalt. However, the SBS modified asphalt with a high modifier dosage is limited in its application due to its poor storage stability. Gong et al. [13] found that the rubber and additives are effective in increasing asphalt viscosity, elastic property, and resistance to permanent deformation. However, the prepared rubber-modified asphalt must be used as soon as possible because it has poor stability. The HVA prepared by HVM is the most popular in China and Japan. HVM is generally composed of a variety of materials including thermoplastic elastomers, tackifying resins, and plasticizers. TPS modifier is one of the most widely used HVMs [14]. Many research findings and engineering experiences have demonstrated that TPS modifier can significantly improve the performance of HVA [15,16,17], but the price of TPS is too high, which limits its widespread application.

Meanwhile, increasing white pollution urges the use of polymers recycled from waste-plastic to realize a “double win” for the environment as well as performance [18,19]. The major source of harm caused by waste-plastic is pollution of the environment, which endangers the health of people and destroys the cleanliness of cities. Bags, containers, and film products account for the majority of waste plastic in our lives, which is mainly made of polyethylene (PE) of different densities. Many studies have been conducted on the feasibility of using waste plastics as road modifiers to achieve sustainable resource development [20,21]. It is generally accepted that PE can effectively improve the high-temperature performance of asphalt, but its low-temperature and fatigue performance are still controversial [22]. As a result, waste PE can be used as a component in HVM to improve the high-temperature performance of HVA. However, PE is a non-polar crystalline polymer with neatly arranged molecules and large intermolecular forces. If PE is used as a single modifier in modified asphalt, PE particles tend to agglomerate in the asphalt, resulting in a segregation phenomenon. The problem of poor compatibility between PE and asphalt must be solved before application. Hesp [23] proposed a steric stabilization method to improve the compatibility of PE with asphalt. However, the main additive, amino-terminated liquid nitrile rubber (ATBN), has high costs and is not suitable for use in engineering. The research conducted by Gao et al. showed that the polymer melt blending technology allows PE and SBS to react to form co-polymers during the mixing process, thereby realizing the compatibilization process between PE and SBS [24]. In the PE/SBS blend system, the PE particles are uniformly dispersed within the three-dimensional network structure of SBS. Therefore, the PE particles do not agglomerate and are compatible with asphalt. In addition, PE comes in different types, each with a different compatibility with asphalt. Liang et al. [25] found that low-density polyethylene (LDPE) is more compatible with asphalt than other types of PE, and the multiple branches of LDPE improve its compatibility with asphalt. Likewise, additives containing aromatic hydrocarbons can promote the swelling of polymers such as PE and SBS in asphalt, which is advantageous for improving compatibility [26]. The use of aromatic additives should be considered in HVM, such as aromatic oils, furfural extract oils, and petroleum resin. Therefore, Waste-LDPE (WLDPE) compounded with SBS was selected to improve the compatibility of WLDPE with asphalt and, as well as C9-resin and aromatic oil, was added for further improvement.

**Figure 1:**
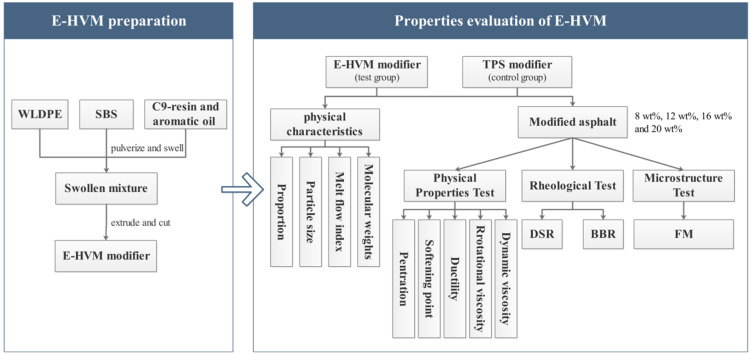
Flowchart of experimental plan procedure in this study.

Based on the above discussion, the purpose of this study is to prepare an environment-friendly HVM (E-HVM) by WLDPE/SBS composite and investigate the effect of the E-HVM modifier on asphalt. The preparation process and physical characteristics of the E-HVM modifier are presented in this paper. Additionally, the properties of HVM modified asphalt were tested, including basic performance tests, rheological property tests, and microstructure tests. The Basic performance tests include conventional indexes of HVM. Through rheological tests, different HVA were characterized by high-temperature rheological properties, fatigue properties, and low-temperature rheological properties. Fluorescence microscopy (FM) was used to observe the dispersion of the modifiers. The flowchart of the experimental plan procedure is shown in Figure 1. It is expected to achieve improved compatibility between WLDPE and asphalt by compounding WLDPE with SBS. This study provides a new solution for recycling waste plastics and has significance for realizing resource recycling.

## 2. Materials and Methods

### 2.1. Materials

SK-70 base asphalt was obtained from the Tongsha Asphalt Technology Co., Ltd., Nantong, China. An overview of the basic properties and chemical composition of the base asphalt is provided in Table 1.

An E-HVM modifier prepared from WLDPE (Nanjing Zhaowei Plastic Product Co., Ltd., Nanjing, China), SBS (Fujian Gulei Petrochemical Co., Ltd., Zhangzhou, China), C9-resin (Kolon Industries Inc., Seoul, Korea), aromatic oil (Royal Dutch /Shell Group of Companies, London, UK), and talcum powder (Guangdong Jade Peak Powder Material Co., Ltd., Foshan, China) by the polymer alloy method [27]. The physical properties of WLDPE, SBS, C9-resin, and aromatic oil are listed in Table 2. Furthermore, the TPS modifier (Taiyu Vietnam Co., Ltd., Hanoi, Vietnam) was used in the study as a test control.

### 2.2. Preparation of E-HVM Modifier 

**Figure 2:**
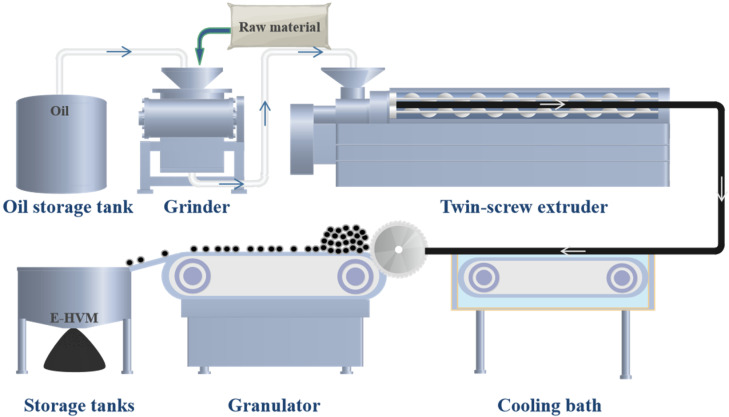
Flowchart of the preparation of E-HVM.

**Figure 3:**
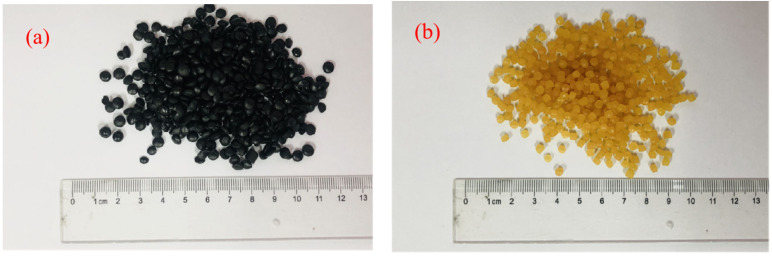
The appearance of two modifiers: (**a**) E-HVM modifier and (**b**) TPS modifier.

The composition of the E-HVM modifier was developed through numerous early exploratory experiments, as shown in Table 3. The E-HVM modifier is prepared in the following manner. Firstly, WLDPE, SBS, and C9-resin are pulverized, and aromatic oil is added during the process. Then, the mixtures are stirred in the grinder for 30 min to ensure that they can swell enough, and talcum powder is added before the next step. Finally, the swollen mixture is injected into an extruder with twin screws for melt mixing with an extrusion temperature of 170 °C and screw speed at 550 rpm. After being extruded, the mixture is cooled in a cooling bath and then cut and sieved by a pelletizer to form the pellet modifier. The flowchart of the preparation of E-HVM modifier is presented in Figure 2, and the appearance of E-HVM modifier and TPS modifier are shown in Figure 3.

### 2.3. Physical Characteristic Tests of Modifiers

The physical characteristic tests are helpful in understanding the basic properties of modifiers and analyzing their modification effect on asphalt: density (ASTM D1505), melting point (ASTM D2117), and MFI (ASTM D1238). Moreover, the molecular weights of modifiers were tested by 1515 gel permeation chromatography (GPC) from Waters, America, and the test solvent was 1, 2, and 4- trichlorobenzene.

### 2.4. Preparation of HVA

The preparation process for HVA was designed based on previous research [15]. The same preparation process was used for two kinds of HVA, and the specific preparation process is as follows. Firstly, the base asphalt is melted, weighed, and poured into the sample barrels. Then, when the base asphalt is heated to 170 °C, the modifier is added gradually by stirring with a glass rod. After the modifier is added, the asphalt and modifier are blended by a high-speed shear machine (FM-300) for 30 min with the temperature at 170 °C and speed at 3000 rpm. Finally, the sheared asphalt is stirred for 1 h at 1500 rpm to obtain HMA samples.

In this paper, the E-HVM modified asphalt prepared with 8 wt%, 12 wt%, 16 wt%, and 20 wt% E-HVM modifiers are marked as EHVM-8, EHVM-12, EHVM-16, and EHM-20, repectively.TPS modified asphalt prepared with 8 wt%, 12 wt%, 16 wt%, and 20 wt% E-HVM modifiers are marked as TPS-8, TPS-12, TPS-16, and TPS-20, respectively. Moreover, three parallel tests were conducted on the same three HVA samples in the paper.

### 2.5. Basic Performance Tests

Conventional tests were conducted to evaluate the basic performance of HVA: penetration (25 °C) (ASTM D5), softening point (R&B) (ASTM D36), ductility (5 °C) (ASTM D113), rotational viscosity (170 °C) (ASTM D4402), dynamic viscosity (60 °C) (ASTM D2171).

### 2.6. Rheological Tests

The DSR-E dynamic shear rheometer (DSR) from Kinexus, Germany, was used to study the medium- and high-temperature rheological behavior of HVA. Strain and stress scanning tests were first performed on individual HVA to ensure that each test was conducted within the linear elastic range [28]. A frequency sweep test using control strain 1% from 20 °C to 60 °C (by 10 °C steps) and the test frequency from 0.1 Hz to 100 Hz was used. The temperature sweep test range was 22 °C to 100 °C (by 6 °C steps) and a frequency of 10 rad/s. Multi-stress creep recovery (MSCR) tests were conducted at 0.1 kPa and 3.2 kPa stress levels with 10 load-unload cycles at 60 °C, each consisting of 1 s creep and 9 s recovery. The Linear amplitude sweep (LAS) test was conducted at 20 °C. The test is divided into two steps. Firstly, HVA should be tested for non-destructive properties by frequency sweep at 0.2 Hz to 30 Hz. Then, HVA was tested for damage characteristics by strain sweep test that ranges from 0.1% to 30%. It is worth noting that when the test temperature is 30 °C and above, 25 mm parallel plate and 1 mm gap are selected for the test. However, when the test temperature is below 30 °C, the 8 mm parallel plate and 2 mm gap are selected for the test.

The TE-BBR bending beam rheometer (BBR) from Cannon, USA, was used to study the low-temperature creep behavior of HVA. Three test temperatures of −12 °C, −18 °C, and −24 °C were selected to obtain and analyze the low-temperature creep rate m and creep stiffness S, which reflect the deformation ability of asphalt in the low-temperature environment [29].

### 2.7. Microstructure Test

XSP-63XD FM from Batuo China was used to observe the microscopic phase behavior of the modifier in the HVA [30]. Polymers can fluoresce under various light sources, so the dispersion of polymers in asphalt can be observed by FM. The samples of different HVA were placed between the slide and coverslip and put into the oven, and the preparation was completed after the asphalt samples were dispersed uniformly between the glass slides. The prepared samples were observed under FM, and microscope images were then obtained.

## 3. Results and Discussion

### 3.1. Physical Characteristics of Modifiers

The physical characteristics of two modifiers are shown in Table 4. Results show that the E-HVM modifier has a higher MFI, but lower density and melting point. Research shows that the dispersion of polymer will be adversely affected by a reduction in MFI [31]. This indicates that the E-HVM modifier will disperse in asphalt more uniformly than TPS modifier. Moreover, the molecular weights of the E-HVM modifier are slightly lower than the TPS modifier, but the PDI is higher. The lower molecular weights of the E-HVM modifier are due to the addition of C9-resin and aromatic oil with a lower molecular weight. The higher PDI indicates a wider molecular weight distribution of the E-HVM modifier. This results may be due to the large difference in molecular weight between the raw materials [27].

### 3.2. Basic Performance of HVA

The effects of different modifiers on the basic performance of HVA are shown in Table 5. Test results show that the basic performance of both types of HVA improved with increasing modifier dosages. In comparison to TPS-modified asphalt, E-HVM-modified asphalt has a higher softening point, 60 °C dynamic viscosity, and 170 °C rotational viscosity at the same dosage from 12 wt% to 20 wt%. Nevertheless, the ductility and penetration of E-HVM-modified asphalt are lower than those of TPS-modified asphalt. So, the E-HVM modifier improves the high-temperature performance of HVA but is slightly worse in terms of workability and low-temperature deformation.

The index requirements in Table 5 are from the Chinese porous asphalt pavement specification (JTG/T 3350-03-2020) [32]. Comparing the test results with the index requirements, it can be found that the E-HVM modifier can meet the technical requirements at 12 wt%, while the TPS modifier can meet the requirements at 16 wt%. The E-HVM modifier requires a lower minimum dosage than TPS modifiers. Therefore, the E-HVM modifier can reduce the amounts of modifiers on the basis of ensuring HVA performance, which may reduce the cost of HVA.

### 3.3. Rheological Properties of HVA

As a non-Newtonian fluid [33], HVA exhibits visco–elastic–plastic properties that vary with test temperature. Conventional tests cannot accurately represent the properties of HVA [34]. To evaluate the rheological properties of different HVAs more comprehensively, DSR was used to test them under different test temperatures and loading modes. In this section, the frequency scan test, temperature scan test, MSCR test, LAS test, and BBR test were used to evaluate the rheological properties of the two types of HVA.

#### 3.3.1. Frequency Sweep Test Result

**Figure 4:**
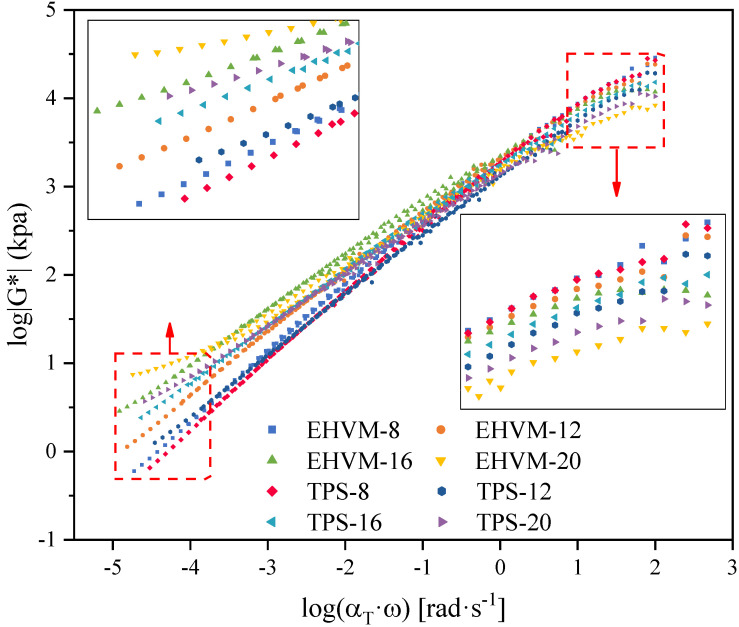
Master curves of different HVA at 20 °C (referenced temperature).

Direct measurement of the viscoelastic properties of asphalt over a wide frequency and temperature range would be time-consuming and extremely demanding on the hardware facilities. According to the time–temperature equivalence principle, decreasing the test frequency has a similar effect as increasing the experimental temperature on the rheological properties of asphalt [35]. Based on the Williams–Landel–Ferry (WLF) equation in Equation (1) [36] and the sigmoidal model in Equation (2) [37], the master curves of different HVA at the 20 °C were established. As is shown in Figure 4, the master curves fit better, and the frequency is increased by several orders of magnitude compared to the test frequency. The high-frequency region corresponds to low temperature, while the low-frequency region is equivalent to high temperature. In the low-frequency region, the complex modulus G* of different HVA is better discriminated than in the high-frequency region. The G^*^ of HVA increased with the increase of modifier dosage, and a higher G* was observed for E-HVM modified asphalt with the same dosage as for TPS modified asphalt. The higher G^*^ indicates a better resistance to deformation of the material [25]. Therefore, The E-HVM modifier improved the high-temperature deformation resistance of HVA better than TPS modifier. This can be attributed to the more stable three-dimensional network structure of WLDPE/SBS composite [38]. There is a reduction in the difference between G* of different HVA as the frequency increases, which indicates that the two modifiers have a greater impact on the performance of HVA at high temperatures than at low temperatures.

(1) $$Log\alpha_{T}=-\frac{C_{1}\left(T-T_{r}\right)}{C_{2}+\left(T-T_{r}\right)},$$

where $C_{1}$ and $C_{2}$ are fitting parameters, T is test temperature, and $T_{r}$ is reference temperature.

(2) $$Log\left|G^{*}\right|=\theta +\frac{\alpha -\theta }{1+e^{\beta +\gamma \cdot logf_{r}}},$$

where $G^{*}$ complex modulus, θ is value of lower asymptote of dynamic modulus, α is value of upper asymptote of dynamic modulus, and β and γ are shape factors.

#### 3.3.2. Temperature Sweep Test Result

**Figure 5:**
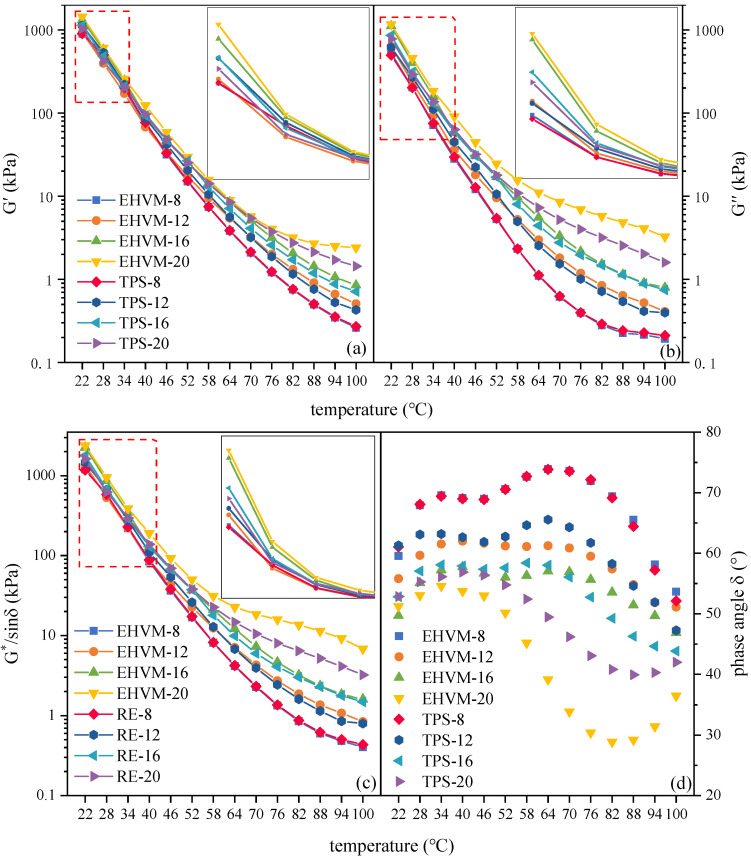
Temperature sweep test results for different HVA: (**a**) storage modulus G′; (**b**) loss modulus G″; (**c**) rutting factor G*/sinδ; and (**d**) phase angle δ.

Temperature sweep test was conducted to investigate the temperature dependence of viscoelastic properties of different HVA. With changing test temperatures, Figure 5 displays the variation in storage modulus G′ value, loss modulus G″ value, rutting factor G*/sinδ value and phase angle δ value of different HVA. G′ value, G″ value, G*/sinδ value and δ value reflect the stiffness, rutting resistance and viscoelastic properties of the HVA [10]. Figure 5a,b show that the G′ and G″ of HVA decreased with increasing temperature. This was due to polymer molecular chains in HVA moving more rapidly with growing temperature, leading to stronger intermolecular forces, which reduces the G′ and G″ of HVA [39]. Moreover, the G′ and G″ values of different HVA differed less in the medium-to-high temperature interval (22 °C to 58 °C) and exhibited a good linear relationship in semi-logarithmic coordinates. As the test temperature increases, the rate of change of G′ and G″ values decrease and tend to level off. It indicates that different HVA exhibit good high-temperature stability, and its complex internal structure is not destroyed by higher temperatures. The G*/sinδ values show a similar pattern to the G′ values and G″ values. Observing Figure 5d, the δ values of different HVA followed a similar pattern, and the δ values of HVA increased as temperature increased. When the temperature exceeds 64 °C, the δ value of the HVA with modifier admixture of 16 wt% and below gradually decreases. The HVA with 20 wt% dosage displayed this interesting phenomenon at a lower temperature than the other HVA. A possible explanation for this phenomenon is that the E-HVM modifier and TPS modifier consist of multiple polymers, and the polymers become more active as the temperature rises, improving the viscoelastic properties of HVA [28].

Observing the whole test temperature range, with the addition of modifier admixture, the G′ value, G″ value, and G^*^/sinδ value of the HVA increased, but the δ value decreased, indicating that the addition of a modifier increased the high-temperature stability of HVA. Chen et al. [40] also found a similar conclusion that the viscosity of asphalt increases as the HVM dosage increases, thereby increasing its resistance to high-temperature deformation. Comparing the two HVAs at the same modifier dosage, the E-HVM modified asphalt has higher G′ value, G″ value, and G*/sinδ value than TPS modified asphalt. This means the performance of the E-HVM modified asphalt is better at high temperatures. Thus, the E-HVM modifier has a greater effect on the high-temperature stability of HVA but may negatively impact the deformability of HVA at low temperatures and cause low-temperature cracking. Overall, the E-HVM modifier imparted better rutting resistance to asphalt when dosed at 12 wt% or higher than the TPS modifier, which confirmed the findings of the frequency sweep test.

#### 3.3.3. Multi-Stress Creep Recovery Test Result

**Figure 6:**
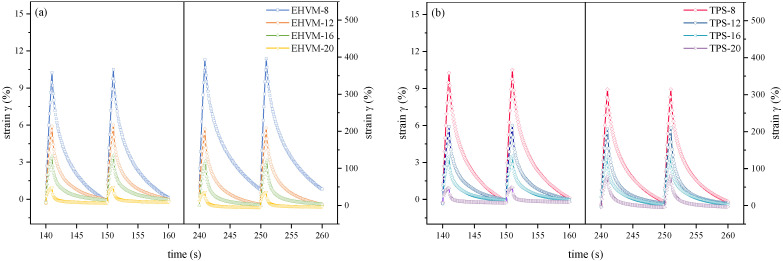
The accumulated strains of different HVA in 5th, 6th, 15th, and 16th cycle: (**a**) E-HVM modified asphalt and (**b**) TPS modified asphalt.

MSCR test is designed to mimic actual pavement conditions [41]. This study used two stress levels to simulate different loading or traffic conditions: 0.1 kPa and 3.2 kPa. The irrecoverable flexibility $J_{\mathrm{nr}}\left(\tau \right)$ and creep recovery R(τ) obtained from MSCR test were used to evaluate the high-temperature resistance of asphalt materials to permanent deformation. Figure 6 shows the cumulative strain curves of different HVAs under different stress levels. The stress level has a significant impact on shear strain generation of HVA. The strain of HVA generated at high stress levels was tens of times higher than that at low stress levels, as well as having a higher cumulative strain. This is the reason why the road structure is prone to rutting and other diseases under heavy traffic. The accumulated strain curves of the two types of HVA show a similar pattern. The higher the modifier dosage, the lower the strain generated at the end of the creep phase of HVA at different stress levels, as well as the lower the irrecoverable strain generated after the creep-recovery cycle. This is because the network structure of HVA is more serried and intersects with itself as the modifier dosage increases; the structure becomes more stable, therefore improving high-temperature performance. Consequently, the two modifiers can both enhance the high-temperature deformation resistance of HVA.

**Figure 7:**
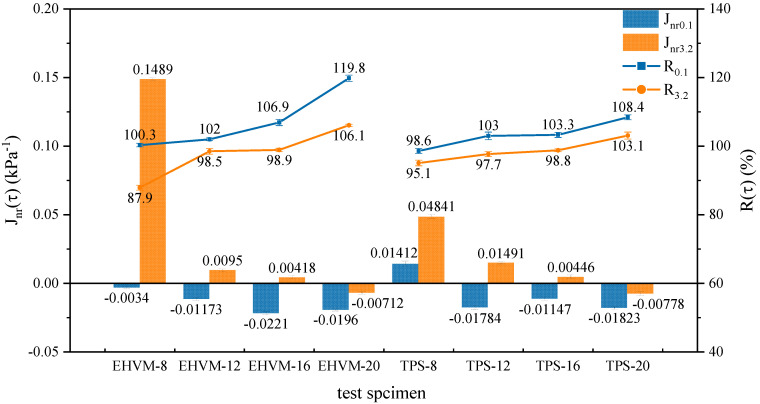
MSCR test results for different HVA. Note: In the figure, the error bars represent the standard deviation of three parallel experiments. The same in the following figure.

Figure 7 shows the specific values of $J_{\mathrm{nr}}\left(\tau \right)$ and R(τ) for different HVA. At 0.1 kPa stress level, the $J_{\mathrm{nr}0.1}$ of the HVA is less than 0, and the $R_{0.1}$ is higher than 100%. This means that the 0.1 kPa stress level cannot cause permanent deformation of the HVA. With the external load removed, the HVA deforms in the opposite direction under the rebound force and may eventually return to its original state. Comparing the $J_{\mathrm{nr}}\left(\tau \right)$ values and R(τ) values of E-HVM modified asphalt and TPS modified asphalt at a 3.2 kPa stress level, it can be found that the index values of the two types of HVA are relatively close. EHVM-20 shows a decrease of 174.9% in $J_{\mathrm{nr}3.2}$ value and an increase of 7.7% in $R_{3.2}$ compared to EHVM-12, while TPS-20 shows a decrease of 152.2 % in $J_{\mathrm{nr}3.2}$ value and an increase of 5.5% in $R_{3.2}$ compared to EHVM-12. At the same dosage from 12 wt% to 20 wt%, the E-HVM-modified asphalt has a lower $J_{\mathrm{nr}3.2}$ value than that of the TPS-modified asphalt and higher $R_{3.2}$. It indicates that the E-HVM-modified asphalt exhibits less deformation under dynamic shear loading and has better elastic-recovery properties. This was because WLDPE and C9-resin in the E-HVM modifier improved the rheological properties of the HVA, while the SBS imparted elastic properties to the HVA, so that enhancing the overall resistance to deformation capacities and elastic recovery capacities [42]. Therefore, by applying E-HVM modifiers, HVA can have better elastic recovery and be less prone to permanent deformation under cyclic loading at high temperatures.

#### 3.3.4. Linear Amplitude Sweep Test Result

**Figure 8:**
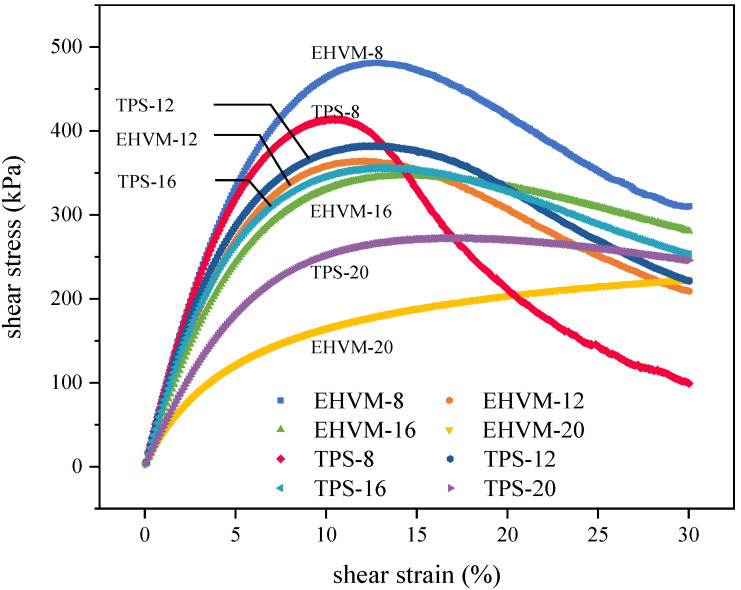
Plot of stress versus strain in the LAS test.

In order to investigate the effect of the E-HVM modifier on the low- and medium-temperature fatigue performance of HVA, different HVA was tested by LAS test and the stress vs strain curve is shown in Figure 8. It was found that the HVA with a lower modifier dosage gradually yielded, whereas the HVA with higher modifier dosage had the mechanical properties of elastomers and remained basically unchanged when the stress reached its maximum. So, the addition of the modifier can improve the elastic properties of HVA at low and medium temperatures and ensures the internal structural integrity of the HVA even under large shear strains. In the LAS test, peak stress does not necessarily reflect fatigue properties of the HVA, but it can reflect stiffness. As the modifier dosage increases, the peak stress of the HVA tends to decrease. The polymer in the modifier improves the deformation ability of asphalt at low and medium temperatures. At the same modifier dosage, the E-HVM-modified asphalt exhibits higher peak stress compared to the TPS-modified asphalt, indicating that the E-HVM modifier makes HVA more rigid, preventing it from deforming.

**Figure 9:**
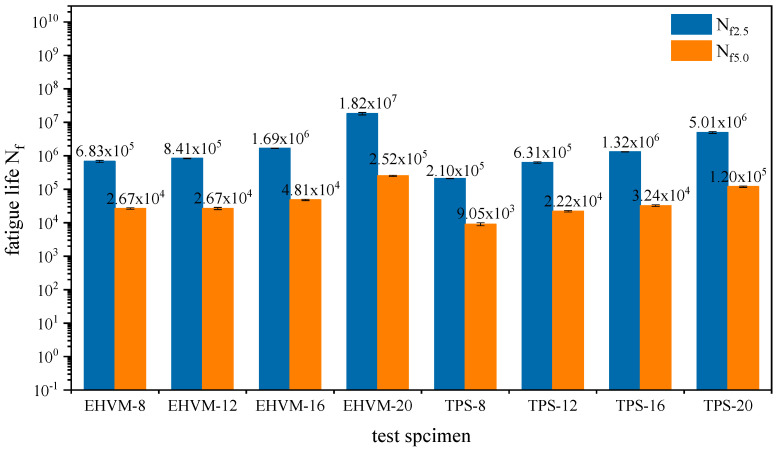
Fatigue life from the LAS test at 2.5% and 5.0% strain levels.

The VECD method was used to calculate the fatigue life for different HVAs at different shear strain levels [43]. Based on two typical strain levels (2.5% and 5%), Figure 9 shows the fatigue life of different HVA. Compared to low-strain levels, the fatigue life of the same kind of HVA at high-strain levels is much shorter. When comparing fatigue lives of different HVA, it was found that increasing modifier dosages could prolong fatigue lives. It is because the addition of the modifier improves the elastic properties of HVA at medium and low temperatures, causing the HVA to generate less stress under the same shear strain, which is beneficial to its fatigue life. In addition, the E-HVM-modified asphalt had longer fatigue life than the TPS-modified asphalt at the same dosage. Specifically, when the shear strain is 2.5%, the N_f_ of EHVM-20 is 3.63 times as long as that of TPS-20, with a shear strain of 5.0%, the N_f_ of EHVM-20 is 2.1 times as long as that of TPS-20. In other cases, the E-HVM-modified asphalt has a slightly longer fatigue life than the TPS-modified asphalt. This is because the E-HVM modifier containing 55 wt% SBS, the network structure of the SBS modifier provides HVA with elastic properties in medium- and low-temperature environments, which is beneficial for improving the fatigue life of HVA [44]. Therefore, for HVA fatigue performance improvement, two types of modifiers are both effective, but the E-HVM modifier works better than the TPS modifier.

#### 3.3.5. Low-Temperature Bending Beam Rheology Test Result

**Figure 10:**
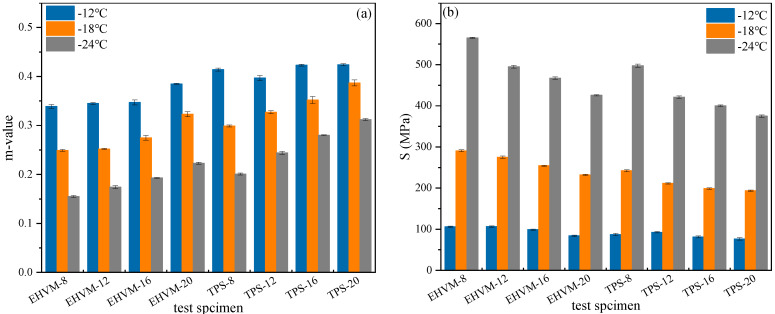
Rheological test results of low-temperature bending beams at different test temperatures: (**a**) Creep rate; (**b**) Creep modulus of stiffness.

To understand the low-temperature rheological properties of HVA, the low-temperature creep behaviors of different HVA were tested by a BBR test under 60 s loading time at −12 °C, −18 °C, and −24 °C. The creep rate m and creep modulus of stiffness S were obtained by testing. The creep rate m reflects the rheological impulse properties of the material, and the creep modulus of stiffness S reflects the toughness and resistance to deformation of the material [45]. Figure 10 shows that the test temperature decreased, the m value decreased, but the S value increased. This indicates a decrease in the low-temperature deformation capacity of the material and an increase in the likelihood of low-temperature cracking. With the increase of the modifier dosage, the m value of the HVA increases, and the S value decreases. This is because the mechanical properties of HVA at low temperatures are similar to those of elastomers. The polymer in the modifier has a better low-temperature deformation capability than the base asphalt. The addition of the modifier reduces the overall low-temperature stiffness of the HVA and enhances the low-temperature deformation capacity of the HVA, which is beneficial for low-temperature crack resistance. When comparing the results of different HVAs, the TPS-modified asphalt showed larger m value and smaller S value than the E-HVM-modified asphalt at the same test temperature and modifier dosage. This shows that TPS-modified asphalt is less likely to crack under loads when temperatures are low, showing better stress-relaxation ability. This is because the WLDPE increases the rigidity of HVA at low temperatures, which reduces the asphalt-binder deformation ability at low temperatures. Therefore, the E-HVM modifier is not as good as the TPS modifier at improving the low-temperature performance of HVA.

### 3.4. Microstructural Characterization of HVA

**Figure 11:**
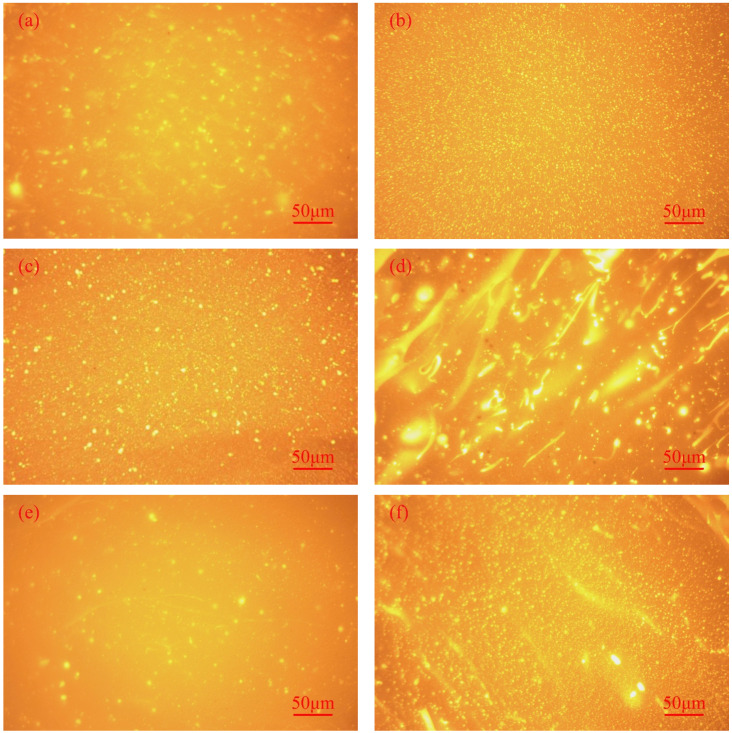

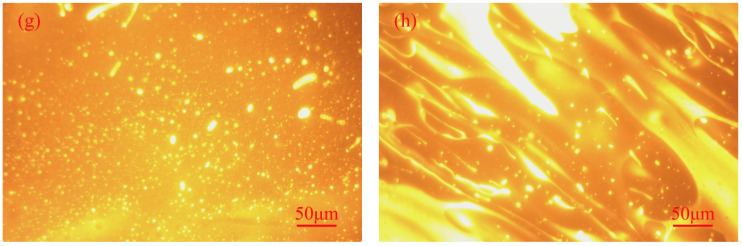
Fluorescence microscopy of different HVA: (**a**) EHVM-8; (**b**) EHVM-12; (**c**) EHVM-16; (**d**) EHVM-20; (**e**) TPS-8; (**f**) TPS-12; (**g**) TPS-16; (**h**) TPS-20.

The dispersion state of the modifier in the asphalt determines the performance of the HVA. Each modifier in the asphalt can be visually reflected in FM images. Figure 11 shows the fluorescence images of different HVAs, the dark part of the figure is the asphalt phase, and the glowing one is the polymer phase. According to Figure 11a,e, when the modifier dosage is 8 wt%, the polymer phase is severely separated from the asphalt phase, and the polymer is free in the asphalt phase. Therefore, the mixture system is unstable. As a result, the lower-modifier dosage is not capable of forming a stable homogeneous state. For this reason, the HVA properties will not meet the requirements when the modifier content is 8 wt%. Compared with the EHVM-8 and TPS-8, the polymer-dispersion state of the two types of HVA significantly improved with modifier admixture. Under the action of high-speed shear, the modifier is uniformly dispersed to form a dense and uniform mesh. However, it was found that both types of HVA appeared large polymer particles when the modifier dosage was 20 wt%. This is due to the fact that the internal asphalt structure began to change from asphalt to polymer, which negatively impacts the modification effect of the modifier. So, both modifier dosages should be controlled below 20 wt% in the subsequent application. Based on the comparison of two types of HVA with the same modifier dosage, the E-HVM-modified asphalt has a more uniform dispersion of polymer particles, whereas the TPS-modified asphalt has a partial agglomeration of polymers. Polymer particles with smaller sizes contribute more to asphalt performance improvement. It is also noteworthy that the poor compatibility of PE with asphalt has been mentioned in past reports [46]. In this study, the agglomeration of WLDPE particles was effectively prevented by melting and co-blending SBS, C9-resin, and aromatic oil with WLDPE. Improvement in WLDPE compatibility with asphalt made it easier for an E-HVM modifier to be uniformly dispersed in asphalt and gave full play to the modifying effect of the modifier.

## 4. Conclusions

In this study, an E-HVM modifier was prepared by a WLDPE/SBS composite, and its effects on the rheological properties and microstructure of asphalt were investigated. Based on the results and discussion, the following conclusions were obtained.

(1) Compounding WLDPE with SBS, the compatibility problem of WLDPE is solved, and the modification effect of the modifier is also enhanced. The E-HVM modifier has higher MFI but a lower density and melting point. Moreover, the E-HVM modifier has lower molecular weights and higher PDI than TPS modifier. Therefore, the E-HVM modifier has better compatibility with asphalt, which makes it easier for E-HVM to disperse in asphalt with smaller average particle sizes during HVA preparation. Therefore, the E-HVM modifier has a better modification effect on asphalt than the TPS modifier.
(2) All basic performances of HVA were improved by adding the E-HVM modifier. At the same dosage from 12 wt% to 20 wt%, the E-HVM-modified asphalt had a higher softening point, 60 °C dynamic viscosity, and 170 °C rotational viscosity than those of the TPS-modified asphalt but lower ductility and penetration. Meanwhile, according to Chinese specification (JTG/T 3350-03-2020), the minimum dosage of the E-HVM modifier was 12 wt%, which is smaller than that of TPS modifier.
(3) According to the results of high-temperature performance indexes (G*, G*/sinδ, $J_{\mathrm{nr}}\left(\tau \right)$, R(τ), etc.), the E-HVM-modified asphalt is better than the TPS-modified asphalt at the same dosage from 12 wt% to 20 wt%. Furthermore, compared to TPS-modified asphalt, E-HVM-modified asphalt has a higher fatigue life at different strain levels (2.5% and 5.0%). However, the E-HVM-modified asphalt has worse low-temperature properties than the TPS-modified asphalt, which is manifested by lower penetration, ductility and creep rate m, and higher creep stiffness modulus S.
(4) Compared to the TPS modifier, the E-HVM modifier is dispersed more uniformly in asphalt at the same dosage. However, the agglomeration and crosslinking of the E-HVM modifier in asphalt are serious at the dosage of 20 wt%, which affects the stability of the blend system. All factors considered, the reasonable dosage of E-HVM modifier is from 12 wt% to 16 wt%.

## Figures and Tables

**Table 1:** Testing results of basic properties of SK-70 base asphalt.

Properties		Unit	Measured Value	Specifications
Penetration (25 °C, 100 g, 5 s)		0.1 mm	70.9	ASTM D5
Penetration index (PI)		/	−0.9	ASTM D5
Softening point (R&B)		°C	47.5	ASTM D36
Ductility (10 °C, 5 cm/min)		cm	49.7	ASTM D113
Ductility (15 °C, 5 cm/min)		cm	101	ASTM D113
Brookfield viscosity (60 °C)		Pa·s	198	ASTM D4402
After RTFOT aging	Mass loss	%	0.03	ASTM D2872
	Penetration ratio	%	62.2	ASTM D5
	Ductility (10 °C, 5 cm/min)	cm	7.6	ASTM D113
	Ductility (15 °C, 5 cm/min)	cm	31.5	ASTM D113
Saturates		wt%	11.71	ASTM D4124
Aromatics		wt%	29.95	
Resins		wt%	49.01	
Asphaltenes		wt%	9.33	

**Table 2:** Physical properties of WLDPE, RHDPE, SBS, C9-resin, and aromatic oil.

WLDPE	Value	SBS	Value	C9-Resin	Value	Aromatic Oil	Value
Density (g/cm^3^)	0.96	Density (g/cm^3^)	0.94	Density (g/cm^3^)	0.99	Density (g/cm^3^)	1.05
Melting point (°C)	107.5	MFI (g/10 min)	5.1	Mw (daltons)	1753	100 °C viscosity (mm²/s)	27.5
MFI (g/10 min)	2.14	Tensile strength (MPa)	15.1	Softening point (°C)	101.7	Aromatic content (%)	87.6
Tensile strength (MPa)	29.4	Elongation (%)	720.6	Tg (°C)	81	Flash point (°C)	265

Note: MFI-melt flow index; Mw-weight average molecular weight.

**Table 3:** Material composition of E-HVM.

Raw Materials	WLDPE	SBS	C9-Resin	Aromatic Oil	Talcum Powder
Weight percentage (wt%)	15	55	15	14.5	0.5

**Table 4:** The physical characteristics of two modifiers.

Properties		Unit	E-HVM Modifier	TPS Modifier
Density		g/cm^3^	0.91	0.94
Melting point		°C	117.4	124.5
MFI		g/10 min	12.1	10.7
Molecular weight distribution	Mn	daltons	198,723	213,214
	Mw	daltons	267,785	274,324
	Mz	daltons	164,127	172,132
	PDI	/	1.3475	1.2866

Note: Mn-average molecular weight; Mw-weight average molecular weight; Mz-Z average molecular weight; PDI-polymer dispersity index.

**Table 5:** Basic performance of HVA.

Properties	Unit	Index Requirement	E-HVM/wt%				TPS/wt%			
			8	12	16	20	8	12	16	20
Penetration (25 °C, 100 g, 5 s)	0.1mm	≥40	54.2	48.7	44.2	41.8	52.2	49.8	45.3	42.5
Std			0.3055	0.1528	0.3606	0.5508	0.2517	0.2517	0.1528	0.1155
Softening point (R&B)	°C	≥80	73.8	93.4	99.5	105.7	79.7	92.7	96.4	100.8
Std			0.5859	0.2000	0.1155	0.4359	0.1155	0.6658	0.1528	0.4509
Ductility (5 °C, 5 cm/min)	cm	≥30	33.8	36.0	41.2	45.2	36.5	47.1	52.6	57.1
Std			1.4844	0.7638	0.1528	1.5535	0.4509	1.2097	0.2000	0.4000
Dynamic viscosity (60 °C)	Pa·s	≥50,000	4285	82,982	119,870	319,618	7119	32,525	74,475	218,013
Std			821.6	668.9	664.9	13551.7	512.0	1255.9	1254.6	4218.5
Rotational viscosity (170 °C)	Pa·s	≤3.0	0.480	0.903	1.335	1.838	0.379	0.600	0.874	1.345
Std			0.0045	0.0101	0.0098	0.0224	0.0036	0.0160	0.0064	0.0299

## Data Availability

Not applicable.

## Abbreviations

ATBN	amino-terminated liquid nitrile rubber
BBR	bending beam rheometer
DSR	dynamic shear rheometer
E-HVM	environment-friendly high-viscosity modifier
FM	fluorescence microscopy
GPC	gel permeation chromatography
HVA	high-viscosity asphalt
HVM	high-viscosity modifier
LAS	Linear amplitude sweep
LDPE	low density polyethylene
MFI	melt flow index
MSCR	Multi-stress creep recovery
Mn	average molecular weight
Mw	weight average molecular weight
Mz	Z average molecular weight
PDI	polymer dispersity index
PE	polyethylene
SBS	styrene–butadiene–styrene
TPS	Tafpack-Super
WLDPE	waste low density polyethylene
WLF	Williams–Landel–Ferry
